# Items

**Published:** 1907-08

**Authors:** 


### ITEMS.

Battle and Company, Saint Louis, have issued pamphlet, No. 2, of the series of dislocations illustrating unilateral dislocation of cervical vertebra, which will be sent free to physicians on application.

Substitution Knocked Out in New York.—The Page bill making substitution a penal offense passed the New York Legislature after a bitter fight against it by some of the large drug concerns, who are notorious substitutors and imitators. The credit for the passage of this much-needed measure is largely due to Charles Roome Parmele of the Parmele Pharmaceutical Company New York. I will publish it in next issue with some "remarks." We congratulate the New York profession and Mr. Parmele on the passage of this righteous act.—*Texas Med. Jour.*

The bill has become a law, Governor Hughes having signed it after a hearing. We join our Texas contemporary in congratulating Mr. Parmele. The new antiseptic, germicide, and disinfectant, Chinosol, of which the Parmele Pharmacal Company is the sole licensee, is presented in our advertising columns for the first time in this issue.

---

What is the most common instrument for carrying out the death sentence? It appears to be the guillotine, which is employed publicly in France, Belgium, Denmark, Hanover and two cantons of Switzerland, and privately in Bavaria, Saxony and also in two cantons of Switzerland. The gallows comes next, says *The Chicago News,* and is favored publicly in Austria, Portugal and Russia, and privately in Great Britain and the United States. Death by the sword obtains in fifteen cantons of Switzerland, in China and Russia publicly, and in Prussia privately. Ecuador, Oldenburg and Russia have adopted the musket, all publicly. In China, too, they have strangulation by the cord, and in Spain the garrote, both public. In Brunswick there is death by the axe, and by the electric chair in New York. In Italy there is no capital punishment.

---

From the Simmenthal region of the Canton Berne comes a story which shows that in Switzerland rusticity and good physique do not necessarily go together. The district is famous throughout the Alps for its fine large breed of cattle and its brand of milk, both of which are highly valued. It is otherwise with the human population. They are spoken of as "coffee faced and flat chested." At the last military draft eighteen young Simmenthal mountaineers were called up, and of these all but four were rejected. This result is said to be not at all uncommon in these Swiss valleys, where cattle and milk are the main sources of income. The peasant feeds himself too much on the latter and grudges himself the former.

